# Identifying Early Signs of Aging: Mobility and Functional Impairment in Middle-Aged Adults

**DOI:** 10.1093/geroni/igaf122.233

**Published:** 2025-12-31

**Authors:** Roee Hayek, Shmuel Springer, Rebecca Brown

**Affiliations:** Ariel University, Ariel, HaMerkaz, Israel; Ariel University, Ariel, HaMerkaz, Israel; University of Pennsylvania, Philadelphia, Pennsylvania, United States

## Abstract

Middle age (45 to 64 years) is a pivotal stage of life when early signs of mobility and functional decline appear to emerge. Despite growing evidence of these changes, no comprehensive review has assessed them. This narrative review examined studies that use self-report assessments to investigate mobility and function and their association with chronic health conditions in middle-aged adults. Data from over 200,000 participants from several countries show that the prevalence of mobility and functional impairments varies widely, from 3.7% to 69.8%, with most studies reporting rates between 15% and 25%. Chronic health conditions have a substantial influence on the prevalence of such impairments, with chronic musculoskeletal pain and arthritis having the highest population-attributable risks (PARs) at 15-38%. Multimorbidity (i.e., having two or more chronic conditions) affects 23-33% of middle-aged adults, with a PAR exceeding 40%. Conversely, hypertension has a PAR of only 3%. Self-reported assessments may underestimate mobility impairments due to ceiling effects, as they are often based on difficulties in activities of daily living (ADLs), which are relatively less demanding for middle-aged adults and primarily capture advanced limitations rather than early or moderate impairments. This analysis underscores the importance of early detection through age-specific tools, targeted measures and interventions to address modifiable risk factors, and integrated care models to ensure long-term mobility and independence.

